# Non‐Surgical Management of People With Frozen Shoulder in the National Health Service: A Review of Publicly Available Patient Information Leaflets

**DOI:** 10.1002/msc.70237

**Published:** 2026-06-22

**Authors:** Ashlie Winter, Gareth Whelan, Hayley Carter, Lisa Pitt, Natasha Maher, Stacey Lalande, Chris Littlewood

**Affiliations:** ^1^ University of Salford Salford UK; ^2^ York and Scarborough Teaching Hospitals NHS Foundation Trust York UK; ^3^ University Hospitals of Derby and Burton NHS Foundation Trust Derby UK; ^4^ Calderdale and Huddersfield NHS Foundation Trust Huddersfield UK; ^5^ Airedale NHS Foundation Trust Keighley UK

**Keywords:** adhesive capsulitis, frozen shoulder, NHS, patient information, physiotherapy

## Abstract

**Background:**

Frozen shoulder, also known as adhesive capsulitis, is a common and disabling condition that causes shoulder pain and progressive stiffness. Patient information leaflets (PILs) are produced by UK National Health Service (NHS) Trusts to help patients understand frozen shoulder and treatment options. However, the content and consistency of these PILs and their alignment with national clinical guidance are currently unclear.

**Objectives:**

This study aimed to identify, analyse and describe the non‐surgical management recommendations presented in publicly available NHS Trust PILs for frozen shoulder and to assess their alignment with the National Institute for Health and Care Excellence (NICE) Clinical Knowledge Summary and British Elbow and Shoulder Society (BESS) best practice resources.

**Methods:**

An online search was undertaken by one reviewer to identify publicly available PILs produced by NHS Trusts detailing non‐surgical management of frozen shoulder. Relevant data were extracted and analysed by one reviewer and verified by five reviewers. Descriptive statistics were used to summarise findings.

**Results:**

Thirty‐eight PILs were identified from 38 NHS Trusts with publication dates ranging from April 2013 to March 2025. Considerable variation was observed in the content, including reference to analgesia, activity modification, exercise prescription and corticosteroid injections. No single PIL reflected all key elements recommended in the NICE Clinical Knowledge Summary and BESS best practice.

**Conclusion:**

The findings demonstrate substantial variation in content, frequent misalignment with current national guidance and best practice exercise recommendations. Such variation may limit and may reduce the clarity, consistency and usefulness of information provided to patients.

## 1 Introduction

Frozen shoulder, also known as adhesive capsulitis, is a common and disabling condition that causes shoulder pain and progressive stiffness (Li et al. 2025). It most commonly affects people aged 40 to 70 years and is more prevalent in women than in men (Kingston et al. 2018; Sun et al. 2024). Although frozen shoulder is sometimes described as self‐limiting, symptoms can persist for months or years, significantly affecting the quality of life (Konarski et al. 2021).

In the United Kingdom (UK), the National Institute for Health and Care Excellence (NICE) Clinical Knowledge Summary recommends non‐surgical management for people with frozen shoulder, including an explanation of the diagnosis, activity modification, analgesia, and supervised or home‐based exercise along with early consideration of a corticosteroid injection (National Institute for Health and Care Excellence 2022). Additionally, the British Elbow and Shoulder Society (BESS) has produced a best practice information and exercise video based on evidence, patient experience, and expert clinical guidance, providing advice on pain management and exercises designed to improve or maintain shoulder range of movement (British Elbow and Shoulder Society 2025). Most people are treated non‐surgically; however, if the condition does not improve sufficiently, then further options, including manipulation under anaesthetic or surgical capsular release, might be considered (National Institute for Health and Care Excellence 2022; Rangan et al. 2015).

To support patient care, UK National Health Service (NHS) Trusts produce patient information leaflets (PILs) intended to help patients better understand their condition and treatment options. However, it is not known whether these PILs reflect current national guidance or best practice recommendations, raising concerns regarding the variability in care.

Hence, this study aimed to identify, analyse and describe the non‐surgical management of people with frozen shoulder according to publicly available PILs produced by NHS Trusts, and assess their alignment with the NICE Clinical Knowledge Summary and BESS best practice recommendations.

## 2 Methods

This study was a descriptive review of publicly available PILs produced by UK NHS Trusts relating to the non‐surgical management of frozen shoulder. Ethical approval was not required for this review of publicly available information.

A single reviewer (AW) conducted a pragmatic web‐based search using Google to identify publicly available PILs from the websites of UK NHS Trusts between 03 June 2025 and 04 June 2025. The following search terms were used:

1. Frozen shoulder, NHS
2. Adhesive capsulitis, NHS
3. Stiff shoulder, NHS
4. Contracted shoulder, NHS

Searching continued until two full consecutive pages of search results returned no new PILs. Data were recorded in Microsoft Excel. Any uncertainties regarding eligibility for inclusion were resolved through discussion among the authors (AW and CL).

### 2.1 Inclusion Criteria

PILs were eligible for inclusion if they were published by a UK NHS Trust, were publicly available and contained information related to the non‐surgical management, including reference to exercise prescription, of people with frozen shoulder.

### 2.2 Exclusion Criteria

PILs from sources other than UK NHS Trusts were excluded, including those from non‐NHS organisations treating NHS patients. PILs focusing exclusively on surgical management were also excluded.

### 2.3 Data Extraction

Data were extracted from each PIL by a single reviewer (AW) and inputted to Microsoft Excel. Four additional reviewers (LP, NM, SL, and CL) independently verified the extracted data for accuracy and consistency. Any uncertainties regarding inclusion were resolved through discussion between the reviewing team. The data extraction categories are outlined in Table 1.

**TABLE 1:** Data extraction categories and detailed description.

Data extracted	Description
NHS trust	Which NHS trust published the leaflet?
Date of publication	If available, the date the information was published by the trust?
Name of condition	By what name(s) did the PIL refer to the condition?
Description	Did the PIL describe the condition, and if so, what description was used?
Risk factors	Did the PIL describe any risk factors, and if so, what were they?
Prevalence by age	Did the PIL mention an age that frozen shoulder is most prevalent, and if so, what was the age or age range?
Prevalence by sex	Did the PIL mention if either sex was more at risk of frozen shoulder, and if so, what were the details?
Description of phases	Did the PIL specify phases of the condition and how did it describe them?
Symptoms	Did the PIL specify symptoms, and if so, what were they?
Duration	Did the PIL mention the duration of the condition, and if so, what was this?
Diagnosis	Did the PIL mention method of diagnosis, and if so, what were the details?
Imaging	Did the PIL mention imaging, and if so, what were the details?
Red flags	Did the PIL mention red flags?
Type of analgesia	Did the PIL recommend analgesia, and if so, what type?
Treatment recommendations	Did the PIL recommend specific treatments, and if so, what were they?
Exercise recommendations	Did the PIL include exercise recommendations, and if so, what were they? (includes categories and specific exercises)
Exercise dosage	Did the PIL specify exercise dosage, and if so, what were the details?
Total exercises	What was the total number of exercises on the PIL?
Advice for completing exercises	Did the PIL include any advice around completing exercises? If so, what was this advice?
Lifestyle advice	Did the PIL include general advice around lifestyle choices, such as smoking and sleep? If so, what was this advice?
Additional advice	Did the PIL include additional advice or education on managing frozen shoulder? If so, what was this advice?
Accessible formats	Did the PIL offer accessible formats, and if so, what were they?

### 2.4 Statistical Analysis

Descriptive statistics (frequencies/percentages) were used to summarise the extracted data. AW undertook the descriptive analysis, and a percentage was verified by HC and CL. All analyses were performed using Microsoft Excel.

## 3 Results

A total of 44 PILs were identified using search terms 1, seven using search terms 2, and one using search terms 4. Search terms 3 did not return any additional relevant PILs. In cases where NHS Trusts split information over multiple PILs, these were combined into a single PIL for data extraction. When duplicate PILs from the same Trust were found, only the most recent version was retained. This resulted in 38 PILs from 38 different UK NHS Trusts included in the analysis. Of these 38, 27 (71%) reported a date of publication, ranging from April 2013 to March 2025, with 20 (53%) of these dated 2020 onwards. Eleven (29%) did not specify a publication date. None of the included PILs gave evidence of patient involvement in their development.

### 3.1 Name of Condition

All 38 PILs (100%) referred to the condition as frozen shoulder. Eleven PILs (29%) referred exclusively to frozen shoulder, whereas 27 (71%) used frozen shoulder in combination with other terms. The most common naming combination was frozen shoulder and adhesive capsulitis, found in 20 PILs (53%). A further two (5%) used frozen shoulder in combination with contracted shoulder. Five PILs (13%) included a mix of three or more terms to describe the condition, including stiff shoulder and capsular contraction.

### 3.2 Description of Condition

Descriptions varied across PILs. The term *inflammation (or inflamed)* was used in 24 PILs (63%), while two further PILs (5%) referred more vaguely to the shoulder being *swollen*. Twenty PILs (53%) referred to capsule thickening or fibrosis. Functional limitations, such as stiffness, tightness, or reduced range of movement, were described in 26 PILs (68%). Six PILs (16%) mentioned pain or muscle guarding, and two (5%) described immune involvement. One PIL (3%) did not describe the condition.

Twenty‐six PILs (68%) reported three distinct frozen shoulder phases. The most common description of phases was ‘freezing, frozen and thawing’, appearing in 14 PILs (37%).

Thirty‐five PILs (92%) referred to an expected duration of frozen shoulder with timeframes varying considerably from a few months to five years. The most common timeframe was ‘up to three years’, which appeared in seven PILs (18%).

### 3.3 Risk Factors

**FIGURE 1:**
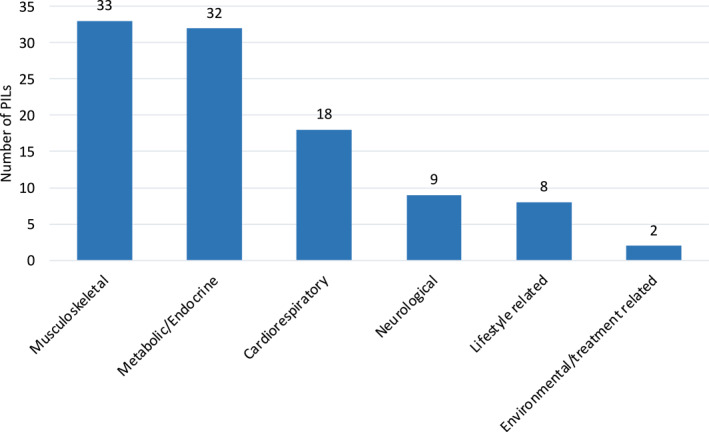
Risk factor categories reported in frozen shoulder PILs.

Risk factors were mentioned in 34 PILs (89%). Musculoskeletal causes were the most frequently cited (33 PILs, 87%), often referring to preceding injury or trauma. Metabolic or endocrine conditions, such as diabetes or thyroid disorders, were mentioned in 32 PILs (84%). Eighteen PILs (47%) reported cardiorespiratory risk factors, nine (24%) reported neurological risk factors, eight (21%) reported life‐style related factors, and two (5%) reported environmental or treatment‐related factors. A breakdown by category is shown in Figure 1.

### 3.4 Prevalence by Age

Thirty PILs (79%) specified an age range at which frozen shoulder commonly develops. Of these, 27 (71%) stated that onset typically occurs over the age of 40 years. The most frequently reported age range was 40–60 years.

### 3.5 Prevalence by Sex

Thirteen PILs (34%) indicated frozen shoulder to be more common in females. Of these, two (5%) provided a female‐to‐male ratio. Twenty‐five PILs (66%) did not specify whether the condition affects one sex more than the other.

### 3.6 Symptoms

Thirty‐seven PILs (97%) described symptoms associated with frozen shoulder, with all referring to pain and stiffness as the main features. Twenty‐one (55%) stated that pain often settles as stiffness increases. Fifteen (39%) noted night pain or disturbed sleep. Thirteen (34%) described pain radiating to the lower arm or hand. Ten (26%) specified which shoulder movements may be affected, including external rotation, abduction, internal rotation, and flexion. Less commonly reported symptoms included muscle wasting (two PILs, 5%), pins and needles (two PILs, 5%), muscle spasm (one PIL, 3%), and neck pain (one PIL, 3%).

### 3.7 Diagnosis and Imaging

Only 13 PILs (34%) indicated how frozen shoulder is diagnosed, all stating it is through patient history and examination. Of these, one PIL (3%) noted that blood tests may be required to rule out other conditions. Only five PILs (13%) mentioned red flags or provided guidance on when to seek further help.

Imaging was referenced in 20 PILs (53%). Fourteen (37%) stated that imaging may be used to exclude other conditions, whilst four (11%) said that it may be needed but did not specify a reason. Two PILs (5%) advised that imaging is not recommended. X‐rays were the most commonly mentioned imaging type, referenced in 12 PILs (32%).

### 3.8 Treatment Recommendations

**FIGURE 2:**
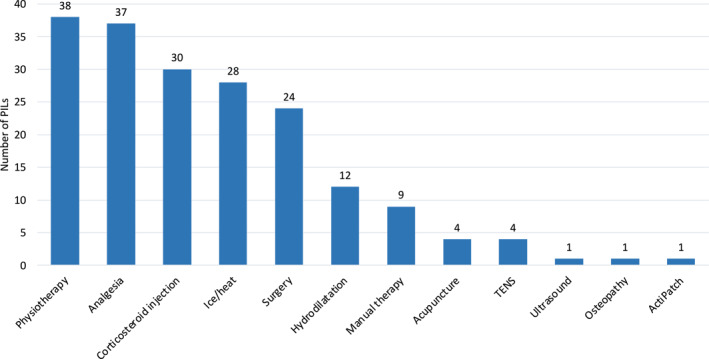
Treatment recommendations in frozen shoulder PILs.

All 38 PILs (100%) recommended physiotherapy, making it the most consistently advised treatment approach. Whilst most PILs suggested combining physiotherapy alongside other approaches, one recommended it as the sole intervention. Analgesia was also highly recommended, mentioned in 37 PILs (97%). Corticosteroid injections appeared in 30 PILs (79%), and the use of heat and ice in 28 PILs (74%). Hydrodilatation was included in 12 PILs (32%), and manual therapy in nine (24%). Acupuncture and TENS were included in four PILs each (11%). Ultrasound, osteopathy, and ActiPatch (a low‐power wearable pulsed shortwave therapy device) were mentioned in one PIL each (3%). Although this review focused on non‐surgical management, it is noted that surgery was mentioned as a treatment option in 24 PILs (63%). All treatment recommendations are outlined in Figure 2.

### 3.9 Type of Analgesia

Of the 37 PILs that recommended analgesia, non‐steroidal anti‐inflammatory drugs (NSAIDs) were the most frequently mentioned, appearing in 24 PILs (63%). Of these, 22 (58%) recommended NSAIDs alongside other analgesics, and two (5%) recommended NSAIDs alone. Unspecified analgesic medication was mentioned in 24 PILs (63%). Paracetamol was recommended in 11 PILs (29%). Codeine and Zapain were each mentioned in one PIL (3%).

### 3.10 Exercise Recommendations

**FIGURE 3:**
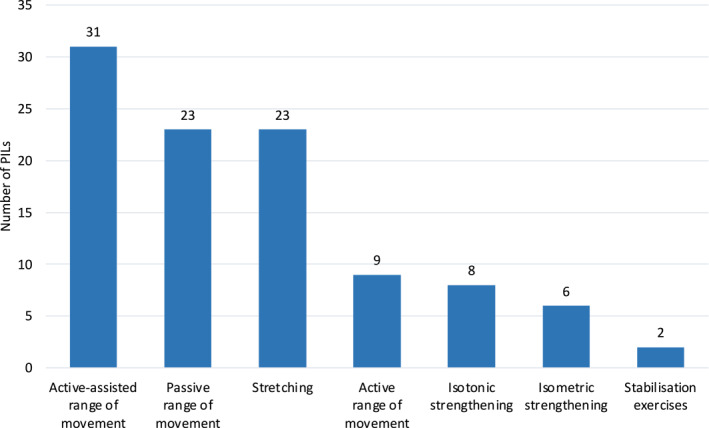
Frequency of exercise types recommended across frozen shoulder PILs.

All 38 PILs (100%) recommended physiotherapy exercises; however, only 31 (82%) provided specific examples. The most commonly recommended exercise type was active‐assisted range of movement, included in all 31 PILs (82%). Passive range of movement and stretching exercises were each included in 23 PILs (61%). Active range of movement exercises appeared in nine PILs (24%). Isotonic strengthening was included in eight PILs (21%), isometric strengthening in six (16%), and stabilisation exercises in two (5%). All exercises focused on the upper body, except for one PIL (3%), which included lower body exercises. These were not included in the analysis. Figure 3 shows the frequency of recommended exercise types.

The most commonly mentioned individual exercises were active‐assisted shoulder flexion (27 PILs, 71%), active‐assisted external rotation (22 PILs, 58%), and shoulder pendulums (21 PILs, 55%). A list of specific exercises within each category can be found in the Table S1.

Among the 31 PILs that provided exercise examples, the number of exercises ranged from two to 21. The most common number of exercises was four, shown in six PILs (16%).

General exercise guidance and advice was included in 33 PILs (87%), even though only 31 gave specific examples. The most common advice was to stop if the exercises became too painful (15 PILs, 39%) and if some discomfort, aching or stretching sensation was normal (14 PILs, 37%). A full summary of exercise guidance is presented in Table 2.

**TABLE 2:** General exercise guidance and advice in frozen shoulder PILs.

General advice for completing exercises	Number of PILs
Do not push through pain; stop if too painful	15
Mild discomfort, aching, or a stretching sensation is normal	14
Modify exercises as needed (e.g., reduce repetitions, range, or intensity)	11
Advice tailored to specific frozen shoulder phases	8
Use ice or heat before or after exercise	8
Analgesia prior to exercise may reduce discomfort and improve tolerance	7
Emphasise movement quality; perform exercises slowly and with control	5
Begin gradually and progress as tolerated	4
See a physiotherapist if pain level is high or if unsure of technique	4
Advice tailored to specific exercises	4
Do not force the movement	2
Early initiation of exercise may support recovery	1
Stop exercise if new symptoms appear	1

### 3.11 Exercise Dosage

Sets: Among the 31 PILs that provided exercise examples, most recommended a single set per exercise. This was either explicitly stated or implied in 25 PILs (66%). One PIL (3%) recommended three sets, and two PILs (5%) included a mix of one and three sets. Three PILs (8%) did not specify the number of sets.

Repetitions: Recommendations for repetitions varied widely. Eight PILs (21%) suggested the same number of repetitions for all exercises, most commonly 10. In 18 PILs (47%), different exercises were assigned different repetition counts. One PIL (3%) advised completing as many repetitions as possible for a specific exercise. Four PILs (11%) did not report any repetitions.

Holds: Hold durations were inconsistently reported and mainly appeared in stretching or isometric strengthening exercises. Reported holds ranged from five to 60 seconds, with five seconds being the most common (4 PILs, 11%). One PIL (3%) advised holding the position but did not specify a duration. Eleven PILs (29%) did not include hold times for any exercises.

Frequency: Exercise frequency varied considerably. Four PILs (11%) recommended performing exercises one to two times per day, four (11%) recommended two to three times per day, and four (11%) suggested three to four times per day. Three PILs (8%) advised completing exercises twice per day, one (3%) recommended three times per day, one (3%) suggested five times per week, and one (3%) advised ‘little and often’. Three PILs (7%) adjusted frequency based on exercise type and one (3%) varied it based on the phase of frozen shoulder. One PIL recommended continuing the exercises for a duration of 12 weeks; no other PILs provided guidance on how long to continue. Eleven PILs (29%) did not give frequency recommendations.

### 3.12 Lifestyle Advice

**FIGURE 4:**
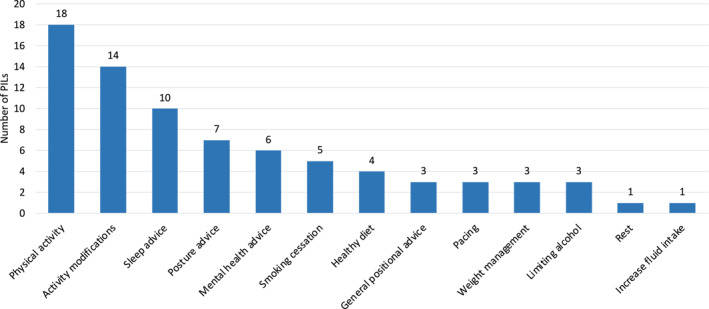
Lifestyle advice from frozen shoulder PILs.

Lifestyle advice was included in 30 of the 38 PILs (79%). The most frequent recommendation was to remain physically active, mentioned in 18 PILs (47%). Fourteen PILs (37%) recommended activity modifications, 10 PILs (26%) provided advice related to sleep, and seven (18%) gave posture advice. Mental health guidance (including stress, anxiety, or relaxation strategies) was included in six PILs (16%). Five PILs (13%) recommended smoking cessation, and four (11%) advised maintaining a healthy diet. Three (8%) gave advice on general positioning, three (8%) mentioned pacing, three (8%) talked about weight management, and three (8%) suggested limiting alcohol intake. One PIL (3%) suggested increasing fluid intake, and one (3%) advised rest. Lifestyle advice is summarised in Figure 4.

### 3.13 Additional Advice

**FIGURE 5:**
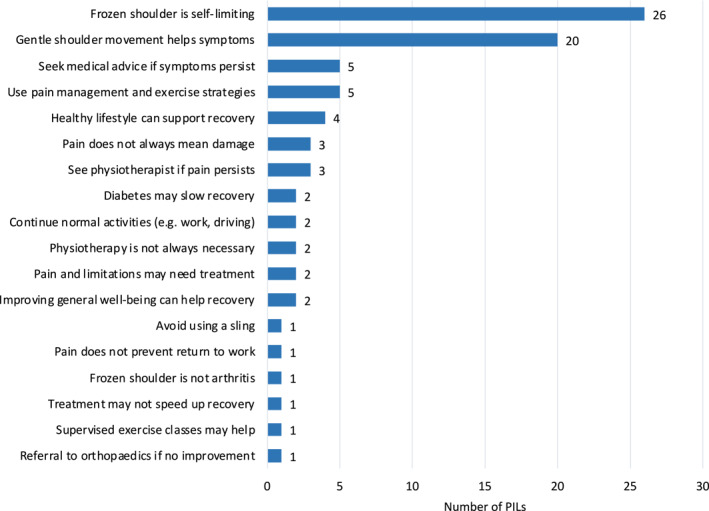
Additional advice from frozen shoulder PILs.

Thirty‐four PILs (89%) included additional education or advice beyond treatment and lifestyle recommendations. The most commonly shared message was that frozen shoulder is self‐limiting, including 26 PILs (68%). This was followed by advice to move the shoulder gently and frequently, as mentioned in 20 PILs (53%). Other themes, such as pain management, diabetes affecting recovery, and the role of physiotherapy, were included less frequently. A full breakdown is shown in Figure 5.

Twenty‐five PILs (66%) included signposting to further information, with the majority directing patients to NHS websites, including NHS Choices and individual Trust pages. Additionally, three PILs (8%) linked to the BESS website, three (8%) to ShoulderDoc, two (5%) to Versus Arthritis, one (3%) to the Chartered Society of Physiotherapy, and one (3%) to the Headspace mental health website.

### 3.14 Accessible Formats

Twenty‐two PILs (58%) mentioned the availability of accessible formats. The most common options were translations into other languages (14 PILs, 37%) and large print (12 PILs, 32%). Braille and audio formats were each offered for eight PILs (21%). Easy Read format was available for five PILs (13%), and British Sign Language for three PILs (8%). High contrast and electronic versions were offered for one PIL (3%). Five PILs (13%) stated that accessible formats were available but did not specify which types.

## 4 Discussion

This review of publicly available NHS Trust PILs showed considerable variation in content and quality, with information often failing to reflect current national guidelines or best practice for frozen shoulder (British Elbow and Shoulder Society 2025; National Institute for Health and Care Excellence 2022).

The NICE Clinical Knowledge Summary recommends a stepped approach to the management of frozen shoulder. This begins with providing information about the diagnosis and likely prognosis, followed by non‐surgical management including analgesia, activity modification, and physiotherapy exercise. If symptoms persist, escalation to a corticosteroid injection or referral to orthopaedics is advised (National Institute for Health and Care Excellence 2022).

While 37 PILs included some description of frozen shoulder, the depth and consistency of information varied. Thirty‐five PILs (92%) referred to an expected duration of the condition, but timeframes ranged from a few months to five years. Such inconsistency may contribute to patient confusion or unrealistic expectations about recovery. Twenty‐six PILs (68%) described frozen shoulder as progressing through three phases, typically termed ‘freezing, frozen, and thawing'. In contrast, NICE uses a two‐phase model of pain‐predominant and stiffness‐predominant phases (National Institute for Health and Care Excellence 2022). These differing frameworks may affect how patients interpret symptom progression and engage with treatments. Several PILs framed the condition as self‐limiting without context, which may underplay the protracted and debilitating nature of symptoms. This lack of clarity could contribute to misunderstandings about recovery, reduced engagement with rehabilitation, and increased health anxiety. Only 14 PILs (37%) included activity modification advice, despite it being a key recommendation. Similarly, while analgesia was mentioned in 37 PILs (97%), recommendations often lacked specificity. Few PILs reflected the NICE Clinical Knowledge Summary's recommended approach of starting with paracetamol and then progressing to NSAIDs or codeine if necessary (National Institute for Health and Care Excellence 2022). Only seven PILs (18%) explained how analgesia could support exercise engagement, which could be an important barrier or facilitator of exercise adherence.

Aligning with the guidance, all PILs recommended physiotherapy exercise; however, there was significant inconsistency in the types of exercises, dosage parameters, and the extent of educational support offered to patients. Although 31 PILs (82%) included specific exercise examples, there was a lack of structure and progression described. A supervised or home‐based exercise programme is recommended early in the course of frozen shoulder treatment, with a duration of 12 weeks advised (National Institute for Health and Care Excellence 2022). Only one PIL (3%) recommended a 12‐week programme, with all others omitting advice regarding duration. Additionally, only one PIL (3%) advised patients to begin exercise early, with three PILs (8%) explicitly advising that physiotherapy should be limited in the early stages.

Corticosteroid injections were mentioned in 30 PILs (79%), which is encouraging given their role in the NICE Clinical Knowledge Summary. A corticosteroid injection is advised early in the course of frozen shoulder if progress with physiotherapy is limited, particularly during the pain‐predominant phase (National Institute for Health and Care Excellence 2022). Although corticosteroid injections were widely recommended, few PILs offered information about early timing, eligibility or potential risks. This lack of detail may limit patient understanding of when and why these injections are appropriate.

Other treatment recommendations, such as heat and ice, appeared in 28 PILs (74%), aligning with NICE guidance, which advises the use of hot packs to manage pain (National Institute for Health and Care Excellence 2022). Manual therapy appeared in nine PILs (24%), and acupuncture was included in four PILs (11%). Whilst these are not core recommendations, they are acknowledged within the NICE Clinical Knowledge Summary as possible components of physiotherapy management (National Institute for Health and Care Excellence 2022). In contrast, interventions such as hydrodilatation, ultrasound, osteopathy, and ActiPatch are not recommended by NICE, yet were included in several PILs. Their inclusion may reflect variation in local clinical practice rather than consistent adherence to national guidance.

The BESS best practice video outlines a structured, phase‐based approach to exercise for frozen shoulder, developed through expert consensus and informed by the UK FROST randomised controlled trial, which used findings to develop a patient exercise booklet that supports consistent, phase‐based care (British Elbow and Shoulder Society 2025; Rangan et al. 2020). The video recommends active‐assisted movement during the early, pain‐predominant phase. Of the 31 PILs that provided exercise examples, all recommended active‐assisted range of movement, reflecting this guidance. However, only one PIL (3%) offered phase‐specific recommendations, indicating limited alignment with the structured approaches advocated by BESS and UK FROST. This also reflects broader inconsistency in how phases are described. Both BESS and UK FROST align with the NICE Clinical Knowledge Summary in describing two phases, pain‐predominant and stiffness‐predominant, whereas the majority of PILs described three phases. This suggests a disconnect between clinical research and the resources made available to patients across NHS Trusts. Notably, however, the BESS and UK FROST recommendations differ in key areas, including exercise quantity, timing, dosage, and progression criteria, even though the former draws on the findings of the latter. Such variation, even among evidence‐based sources, may contribute to the inconsistencies observed in PILs.

In conditions such as frozen shoulder, symptoms can persist for months or years, and patients often have limited understanding of their prognosis or treatment options (Lyne et al. 2022). High‐quality patient information may support psychological wellbeing by reducing uncertainty, whilst also improving patient knowledge and satisfaction (Bolejko and Hagell 2021; Sustersic et al. 2017). Despite this, the PILs included in this review varied considerably in content, format and accessibility. Only 22 PILs (58%) acknowledged accessible formats, despite NHS England's obligation under the Accessible Information Standard to meet diverse communication needs (NHS England 2025).

Overall, the findings of this review suggest that the current provision of frozen shoulder PILs lacks clarity, consistency and alignment with national guidance and best practice recommendations. These findings have several important implications. For clinicians, variation in PILs may result in inconsistent messaging, potentially undermining shared decision‐making and patient confidence. For patients, unclear or conflicting information may lead to uncertainty regarding prognosis, treatment expectations, and a lack of engagement with rehabilitation. There is a need for more research into the development and evaluation of standardised, evidence‐based PILs that align with national guidance and address the accessibility gaps identified in this review. Co‐design approaches involving patients and clinicians may be valuable in ensuring that these resources meet user needs while supporting consistent messaging across services.

### 4.1 Strengths and Limitations

This is the first study to examine non‐surgical management recommendations for people with frozen shoulder as presented in publicly available NHS Trust PILs. However, as the analysis was limited to PILs accessible online, the findings may reflect a selective sample and may not be representative of all those available to patients accessing NHS services (e.g., those not published online). As PILs are periodically updated, the findings represent a snapshot in time and may not reflect subsequent revisions or materials distributed directly in clinical settings. Further, the advice, information and provision of treatment delivered at each hospital may not align with that described in the PIL for that Trust.

## 5 Conclusion

This study found considerable variation in the content and quality of frozen shoulder PILs produced by NHS Trusts, and frequent misalignment with current national clinical guidelines or best practice exercise guidelines. Variation was evident in key areas including core information about the condition, analgesia recommendations, treatment options, exercise prescription, and lifestyle advice. Exercise advice, in particular, was highly inconsistent in selection, dosage, and timing. Such inconsistency may perpetuate variations in care and outcomes. Given the complexity of frozen shoulder and the lengthy recovery process, accessible and accurate patient information is essential. The findings highlight the need for a more standardised, evidence‐based approach to PIL development. Further research into the specific information needs of people with frozen shoulder could help shape resources that align with the national guidance for management and improve the quality of patient information.

## Author Contributions

**Ashlie Winter:** conceptualization, methodology, investigation, data curation, formal analysis, methodology, project administration, visualization, writing – original draft, writing – review and editing. **Gareth Whelan:** data validation, writing – review and editing. **Hayley Carter:** data validation, writing – review and editing. **Lisa Pitt:** data validation, writing – review and editing. **Natasha Maher:** data validation, writing – review and editing. **Stacey Lalande:** data validation, writing – review and editing. **Chris Littlewood:** conceptualization, methodology, validation, writing – review and editing, supervision.

## Funding

The authors have nothing to report.

## Conflicts of Interest

The authors declare no conflicts of interest.

## Supporting information


**Table S1:** Specific exercises recommended in frozen shoulder PILs.

## Data Availability

The data that support the findings of this study are available from the corresponding author upon reasonable request.
