# Supplementary material for: Non‐Surgical Management of People With Frozen Shoulder in the National Health Service: A Review of Publicly Available Patient Information Leaflets
Source: Musculoskeletal Care. 2026 Jun 22;24(2):e70237. doi: 10.1002/msc.70237 (PMC13287040; doi:10.1002/msc.70237)
Supplement: Supplementary file 1 — Table S1: Specific exercises recommended in frozen shoulder PILs. [file MSC-24-e70237-s001.docx]

**Table of specific exercises recommended in frozen shoulder PILs**

| **Exercise type** | **Specific exercise** | **Number of PILs** |
| --- | --- | --- |
| Passive range of movement | Shoulder pendulums | 21 |
|  | Passive flexion | 7 |
| Active-assisted range of movement | Shoulder external rotation | 22 |
|  | Shoulder internal rotation | 5 |
|  | Shoulder flexion | 27 |
|  | Shoulder abduction | 9 |
| Active range of movement | Shoulder external rotation | 2 |
|  | Shoulder abduction | 1 |
|  | Shoulder flexion | 1 |
|  | Repeated internal and external shoulder rotation | 1 |
|  | Finger and wrist flexion and extension | 1 |
|  | Neck rotation, flexion and lateral flexion | 2 |
|  | Shoulder shrugs and rolls | 4 |
| Stretching | Shoulder external rotation stretch | 3 |
|  | Shoulder internal rotation stretch | 15 |
|  | Shoulder flexion stretch | 3 |
|  | Shoulder extension stretch | 1 |
|  | Posterior shoulder stretch | 9 |
|  | Single sided pec stretch (using doorway) | 3 |
|  | Pec stretch/butterfly stretch | 12 |
|  | Inferior capsule stretch | 1 |
| Isotonic strengthening | Weighted overhead press | 2 |
|  | Resisted shoulder external rotation | 4 |
|  | Resisted shoulder internal rotation | 3 |
|  | Resisted shoulder abduction | 3 |
|  | Resisted shoulder flexion | 3 |
|  | Grip strengthening using soft ball squeezes | 1 |
|  | Wall push-ups | 1 |
|  | Side bends (body weight or with dumbbells) | 1 |
| Isometric strengthening | Shoulder external rotation | 6 |
|  | Shoulder internal rotation | 1 |
|  | Shoulder flexion | 2 |
|  | Shoulder abduction | 1 |
|  | Shoulder extension | 1 |
| Stabilisation exercises | Four-point kneeling shifts | 1 |
|  | Scapula squeezes | 1 |
